# Assessing the Retail Food Environment in Madrid: An Evaluation of Administrative Data against Ground Truthing

**DOI:** 10.3390/ijerph16193538

**Published:** 2019-09-21

**Authors:** Julia Díez, Alba Cebrecos, Iñaki Galán, Hugo Pérez-Freixo, Manuel Franco, Usama Bilal

**Affiliations:** 1 Public Health and Epidemiology Research Group, School of Medicine, Universidad de Alcalá, Alcalá de Henares, 28001 Madrid, Spain; alba.cebrecos@uah.es (A.C.); hugo.perez.freixo@gmail.com (H.P.-F.); manuel.franco@uah.es (M.F.); ub45@drexel.edu (U.B.); 2 National Centre for Epidemiology, Instituto de Salud Carlos III (ISCIII), 28029 Madrid, Spain; igalan@isciii.es; 3 Department of Preventive Medicine and Public Health, School of Medicine, Universidad Autónoma de Madrid/IdiPAZ, 28029 Madrid, Spain; 4 Department of Epidemiology, Johns Hopkins Bloomberg School of Public Health, Baltimore, MD 21205, USA; 5 Department of Epidemiology and Biostatistics, and Urban Health Collaborative, Dornsife School of Public Health, Drexel University, Philadelphia, PA 19104, USA

**Keywords:** retail food environment, validity, secondary data, differential exposure, ground-truthing, food outlets, Spain

## Abstract

Previous studies have suggested that European settings face unique food environment issues; however, retail food environments (RFE) outside Anglo-Saxon contexts remain understudied. We assessed the completeness and accuracy of an administrative dataset against ground truthing, using the example of Madrid (Spain). Further, we tested whether its completeness differed by its area-level socioeconomic status (SES) and population density. First, we collected data on the RFE through the ground truthing of 42 census tracts. Second, we retrieved data on the RFE from an administrative dataset covering the entire city (*n* = 2412 census tracts), and matched outlets using location matching and location/name matching. Third, we validated the administrative dataset against the gold standard of ground truthing. Using location matching, the administrative dataset had a high sensitivity (0.95; [95% CI = 0.89, 0.98]) and positive predictive values (PPV) (0.79; [95% CI = 0.70, 0.85]), while these values were substantially lower using location/name matching (0.55 and 0.45, respectively). Accuracy was slightly higher using location/name matching (*k* = 0.71 vs 0.62). We found some evidence for systematic differences in PPV by area-level SES using location matching, and in both sensitivity and PPV by population density using location/name matching. Administrative datasets may offer a reliable and cost-effective source to measure retail food access; however, their accuracy needs to be evaluated before using them for research purposes.

## 1. Introduction

The retail food environment (RFE) is the distribution of food access and defines the context for purchasing decisions [1]. The RFE plays an important role in creating supportive environmental contexts needed for improving population-level dietary patterns and diet-related health outcomes (e.g., obesity) [1,2]. Moreover, the influence of the RFE may be shaped by social environmental factors (e.g., socioeconomic status) [2,3]. Therefore, there is a need to create healthier and more equitable food environments to improve dietary patterns at the population level [4].

Recent systematic reviews have compiled evidence on the role of the RFE in relation to diet [5] and diet-related health outcomes [6,7]. Yet, the resulting evidence on this relationship is mixed, partially because of heterogeneity in the assessment of the exposure, as different studies have used diverse measures to characterize the RFE [8,9,10,11,12]. Studies often rely upon food outlet data either retrieved from ground truthing or from secondary data sources. Ground truthing, the systematic surveying of a territory to identify food outlets, is the ‘gold standard’ for measuring the RFE, but is resource intensive [9,10,11,13]. Thus, research has relied upon secondary data (collected for other purposes that include food outlet location and activity information) as an easier and more accessible method to assess the RFE. These secondary data can be also useful for planning and policy evaluation purposes [14]. Yet, data quality presents an important challenge for food environment research [15].

While most US-based studies have relied on commercial data sources (e.g., InfoUSA) [15], researchers in the UK have used both commercial (e.g., Yellow Pages^®^) and administrative (e.g., local councils’ environmental health departments) data sources [16]. In both settings, administrative records have been more accurate compared to commercial datasets [16,17,18]. Research on the retail food environment in other settings, such as Canada [19,20,21] or Australia [22,23], is also rapidly expanding. This said, retail food environments outside Anglo-Saxon contexts remain understudied. This is important because Southern European settings may face unique food environment issues [24,25,26,27,28]. As compared to Anglo-Saxon settings (like the US), the market share concentration of food retailing activity in Southern European settings has been low [25]. Although supermarkets are increasingly replacing traditional small grocers (e.g., fishmongers), residents continue to shop at small specialized outlets to buy fresh food products [3,29,30]. Yet, these small specialized outlets are less likely to be included in commercial datasets [31,32,33]. Moreover, the concentration of food stores per resident is three times higher in Southern Europe (e.g., Spain) than in the UK [25].

Furthermore, previous studies have suggested that the accuracy of secondary data sources may systematically differ across area-level socioeconomic status or urban form [15,34,35]. These potential differences are key to consider, as they may introduce systematic biases in RFE studies [8]. In Madrid, previous research has shown that the RFE varied by neighborhood-level socioeconomic status [3]. In Copenhagen, another study found no disparities in the RFE regarding socioeconomic nor demographic factors [29]. Both studies used municipal datasets following the standardized statistical classification of economic activities used in the European Union (NACE) [36]. In their systematic review, Fleischhacker et al. suggested validating administrative registries on a case by case basis before using them for research purposes [15]. Yet, little is known about the accuracy of these secondary datasets.

To fill this gap, our aim was to assess the completeness and accuracy of administrative datasets against ground truthing in the city of Madrid (Spain). Secondly, we tested whether this completeness differed by area-level socioeconomic status and population density.

## 2. Materials and Methods

### 2.1. Study Design and Sampling Approach

This study was part of the European Union-funded ‘Heart Healthy Hoods’ project, which examined the urban environment (including the food environment) in relation to residents’ cardiovascular risk in Spain [37]. We did not require any institutional review board for this study, because no human participants were involved.

Our study area was the city of Madrid, with a population of about 3.2 million in 2016. For reference, Madrid was administratively divided into 21 Districts, 128 neighborhoods, and 2412 census tracts in 2016. Census tracts (called census sections in Spain) are the smallest administrative unit for which demographic and socioeconomic data are released in Spain, and have a mean population of 1323 residents per census tract [38]. We used census tracts to define our audit areas.

Our study sample was 42 census tracts scattered around the city. The sampling of these census tracts was based on a combination of socioeconomic characteristics (educational attainment level, immigration, and age distribution) and urban form factors (residential and commercial density).

Details about this sampling strategy have been published elsewhere [39]. Table S1 shows descriptive statistics for these audit areas.

We conducted this study in three phases. First, we collected data on the retail food environment (RFE) through ground truthing of 42 census tracts. Second, we collected data on the RFE using an administrative dataset that covers the entire city of Madrid (*n* = 2412 census tracts). Third, we validated the data obtained from the administrative dataset against the gold standard of ground truthing.

### 2.2. Ground Truthing

In phase 1, we conducted on-field visits in the 42 census tracts. To improve comparability with previous studies, we used an adapted version of the Nutrition Environment Measures Survey for Stores (NEMS-S) for the data collection [26,40]. The NEMS-S tool has shown high inter-rater reliability, test-retest reliability, and face and criterion validity [40,41]. Our adapted NEMS-S survey collected data (for each food outlet) on the business name, street address, hours of operation, outlet type and availability and cost of healthy foods over 11 food groups (fruits, vegetables, nuts, non-alcoholic beverages, bread, cereals and baked goods, milk, dairy products and eggs, oil and butter, rice and pasta, legumes, meat and meat products and fish) [26]. We integrated the NEMS-S audit tool into an easy and freely-accessible web-based app called Open Data Kit [42].

Based on previous studies [3,24], we classified food outlets as described in the classification scheme of Table 1.

During May 2016, two different observers trained in administering the instrument under the supervision of the lead researcher. Between June and July 2016, the three trained observers conducted the data collection, by systematically covering all census tracts on foot, following a map in which the route along the entire census tract was previously defined. Observers examined all food outlets (outlets that were closed were re-visited on another day and time). We collected all data on weekdays, and without notifying or warning the outlet owners or employees to avoid bias.

### 2.3. Secondary Administrative Dataset

In Phase 2, we retrieved food outlet data from an administrative database of the Madrid City Council, which covers all licensed premises citywide. To minimize any temporal mismatch between data sources, we downloaded the data for June 2016. Food outlet data (Censo de Locales y Actividades, Madrid, Spain) are collected by the Department of Statistics for statistical purposes, licensing and inspections; and are freely accessible at the Open Data website (datos.madrid.es). The dataset is updated monthly, and collects name, location (latitude and longitude, along with street address), and type for each premise. Retailer types are coded following the statistical classification of economic activities in the European Community (NACE) [36]. NACE consists of a hierarchical structure, whereby ‘retail trade activities’ are further subdivided into several categories (see Table S2 for more details).

**Figure 1:**
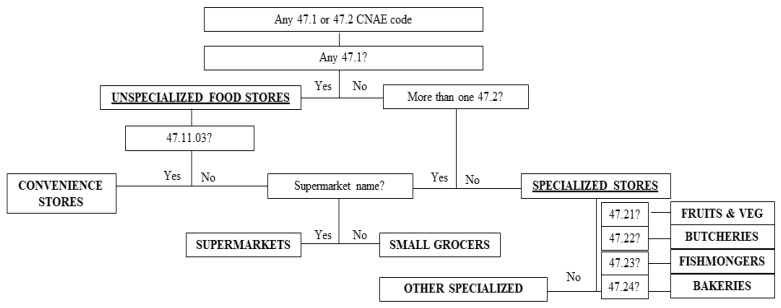
Classification algorithm used to classify food outlets based on a declared code of economic activity and outlet name. We used name recognition to differentiate supermarkets from small grocers, with a list of 60 supermarket names that we obtained from the Yellow Pages^®^ [3].

We developed a classification algorithm based on the economic activity codes of each outlet, and the outlet name that would classify outlets into the same categories as those shown in the classification scheme (see Table 1). We trained this algorithm after ground truthing a different set of 12 contiguous census tracts with a wide variety of outlet types. Further details on this algorithm have been published elsewhere [3,24]. Figure 1 shows the classification algorithm.

### 2.4. Area-Level Socioeconomic Status and Demographic Data

Following previous research, we assessed if the completeness of the administrative data varied geographically by area-level socioeconomic status and population density. [8,43]. To measure area-level socioeconomic status we computed a socioeconomic status (SES) composite index across four domains (education, occupation, living conditions and wealth) suggested for the study of the effect of structural policies on health inequalities in Spain [38]. Specifically, our SES composite index included seven indicators (low education, high education, part-time work, temporary work, manual work, unemployment and average housing prices) [44]. We operationalized the SES measure as a categorical variable using tertiles based on the SES index score distribution across all 42 census tracts: Low (T1), middle (T2), and high (T3). We computed the population density at the census tract level by dividing the number of residents over the land area of the census tract. Population data came from the 2016 municipal population registry. We operationalized this measure as a categorical variable using tertiles based on the distribution across all 42 census tracts.

Each outlet was mapped using the street address and entered into a Geographic Information System, using ArcGIS software 10.3 (ESRI, Redlands, CA, USA). We then overlaid outlet points to census tract boundaries using a spatial join.

### 2.5. Outlet Matching Process

To match outlets in the administrative dataset with outlets found during ground-truthing, we employed two strategies: (1) Location matching (henceforth referred to as liberal matching), and (2) location and name matching (henceforth referred to as strict matching) [45,46,47,48].

To match outlets by location, we used their exact street address (same street name and number). We then reviewed all of the unmatched outlets record-to-record to identify minor errors (e.g., if an outlet was in the same intersection, but the official address listed one street of the intersection instead of the other). Figure S1 shows an example of allowable street names discrepancies. Outlets were matched by name, following previous research [45,46,47], even where business names had minor variations in spelling (e.g., ‘Foody’ and ‘Foodi’) or when they were very similar (e.g., ‘La Plaza’ and ‘La Plaza de Dia’). Table S3 shows all un-matched outlets due to non-allowable discrepancies in outlet names.

### 2.6. Statistical Analysis

To assess the completeness of the administrative dataset, we calculated sensitivity and positive predictive values (PPV). Sensitivity is the proportion of outlets observed during ground truthing that were found in the secondary data.

(1) $$Sensitivity\;Outlets=\frac{observed\;in\;ground\;truthing\;\&\;found\;in\;secondary\;data}{Total\;number\;of\;outlets\;observed\;in\;ground\;truthing}$$

PPV is the proportion of outlets found in the secondary data that were observed during ground-truthing.

(2) $$PPV=\frac{Outlets\;found\;in\;secondary\;data\;\&\;observed\;in\;ground\;truthing}{Total\;number\;of\;outlets\;found\;in\;secondary\;data}$$

We also assessed a possible systematic bias in the completeness of the administrative data according to area-level socioeconomic status and population density. We applied log-binomial regression to assess whether sensitivity and PPV varied by area-level socioeconomic status and population density. We ran two sets of log-binomial regressions for each matching strategy, where the dependent variable was whether the outlet was matched, and the total sample was: (a) For sensitivity, the total number of outlets observed in ground truthing, and (b) for PPV, the total number of outlets found in the secondary data. Area-level SES or population density were the independent variables in this regression. The equation for this model is (for sensitivity and Area-level SES):

(3) $$\begin{array}{cc} log(P\;Matched & =1\;|\;Observed\;in\;ground=1)\\ & =\beta_{0}+\beta_{1}\times MidSES+\beta_{2}\times HighSES \end{array}$$

Where β_0_ is the probability of finding the store in the administrative dataset in low SES areas, β_1_ is the log Prevalence Ratio comparing middle SES to low SES areas, and β_2_ is the log Prevalence Ratio comparing high SES to low SES areas. We jointly tested, using a Wald test, the null hypothesis that both β_1_ and β_2_ were equal to 0, in order to assess whether there were differences in sensitivity or PPV by Area-level SES or population density. To estimate sensitivity and PPV, we computed a linear combination of coefficients (e.g., β_0_ for low SES, β_0_ + β_1_ for middle SES, etc.), then exponentiated this combination to obtain the probability. All log-binomial regression models were estimated using generalized estimating equations (GEE) with robust standard errors, accounting for potential within-census tract dependence between stores.

Finally, we assessed the accuracy of our algorithm to classify outlets by type by using percentage agreement and Cohen’s Kappa statistic measures. We used Fisher’s Exact tests and Clopper-Pearson ‘exact’ 95% confidence intervals (95% CI). We conducted all analyses using STATA/SE 15 (StataCorp, College Station, TX, USA).

## 3. Results

### 3.1. Completeness

**Figure 2:**
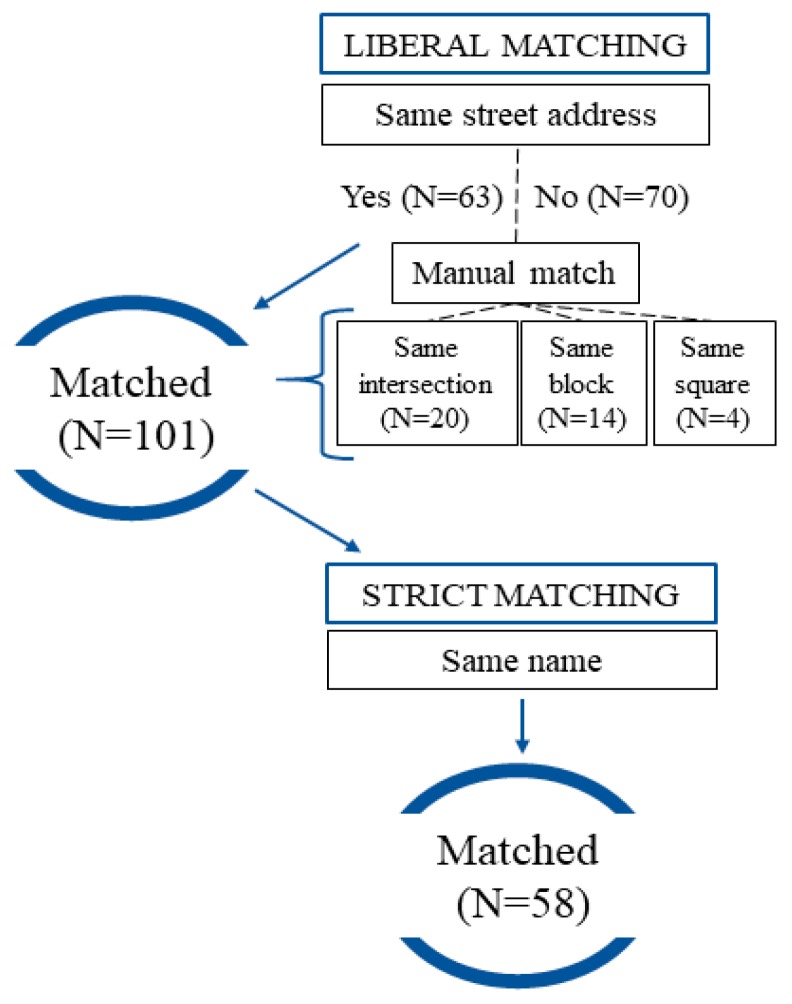
Flowchart illustrating data matching process using both (1) a liberal matching strategy (only by location); and (2) a strict matching strategy (both by location and outlet name).

We observed a total of 106 food outlets during ground truthing, compared to 128 outlets found in the administrative data. Apart from these, the administrative dataset missed 5 of the existing food stores on the field. Using the liberal matching strategy, we matched 101 outlets by location. Out of these, 63 were matched to the same street name and number, while 38 were included after reviewing discrepancies (located in the same intersection, in the same block, or in the same square). Using the strict strategy (location and name), we matched 58 outlets. Figure 2 illustrates this data matching process.

Table 2 shows the results our assessment of completeness. Regarding sensitivity, we found that 95% of the outlets observed during ground truthing could be matched by location to an outlet in the secondary dataset (Sensitivity = 0.95; 95% CI = 0.89, 0.98). With the strict matching criteria, sensitivity dropped to 0.55 (95% CI = 0.44, 0.64). Regarding PPV, we found that 79% of the outlets found in the secondary dataset could be matched by location to an outlet observed during ground truthing (PPV = 0.79; 95% CI = 0.70, 0.85). With the strict matching criteria, PPV dropped to 0.45 (95% CI = 0.37, 0.54).

Table 3 shows the results in terms of the possible systematic bias by area-level socioeconomic status and population density. When applying a liberal matching strategy, we found no evidence for differences in the dataset’s sensitivity, neither across socioeconomic status, nor population density, and no evidence for differences in PPV by population density. However, we found that the PPV was significantly higher in low socioeconomic areas.

When applying a strict matching strategy, we found some evidence for a systematic difference in both sensitivity and PPV by population density. We show the results of the log-binomial regression model used to derive these values in Table S6.

### 3.2. Accuracy

Table 4 shows the results regarding the accuracy of the algorithm to classify correctly the type of outlet in the administrative dataset, by outlet matching strategy. For both matching strategies, we obtained both similar percentage agreement values (71% vs. 77%). In terms of Cohen’s *k* values, we obtained higher values when applying a stricter matching strategy. Table S4 and Table S5 show further details of the differences by outlet type, and by using both matching strategies.

## 4. Discussion

In this study, we found that an administrative dataset was a valid tool in measuring the retail food environment in Madrid (Spain). Specifically, and using a matching strategy based upon location, we found that 95% of the outlets observed during ground truthing were found in the dataset, while 79% of the outlets found in the dataset were observed during ground-truthing. However, we found that making this matching strategy further rely on name (in addition to location) lowered these numbers drastically, down to 55% and 45% respectively. We also found that both strategies had a similar performance in classifying outlets by retailer type (*k* = 0.62 and 0.71, respectively). Last, we found that while there were some systematic biases in the completeness of the administrative dataset by socioeconomic status and population density, these tended to be lower by using the location (liberal) matching strategy.

These findings suggest that the liberal matching strategy (retrieving food outlets using their street address) works best for characterizing the retail food environment (vs. ground-truthing). In this sense, errors in specific business names may not matter to make food retail distinctions (within the same retailer types).

While this idea has been suggested by previous studies [47,48], our study extends previous research because we examined the validity of secondary data sources in a European context and included multiple types of food outlets (e.g., specialized food retailers) [49,50]. This is important, because previous studies have found that independent (non-chain) food outlets are more likely to be missed in commercial datasets [31,32,33,51]. Thus, findings from previous validation studies are difficult to apply to European settings.

In our study, we obtained higher sensitivity and PPV values (based on the liberal matching strategy) than previous US-based research [17,46]. Using a similar matching strategy, coined by Caspi et al. as “lenient matching strategy”, the authors reported average sensitivity values of 62% (and PPV, on average, of 57%) in their validation study across three areas in Minnesota [46]. On the other hand, Liese et al. obtained sensitivity values ranging from 55% to 68% (and PPV values from 78% to 89%) in their validation study (matching stores both by address and name) across eight counties in South Carolina [17]. In Europe, validation studies have been conducted in the UK and in Denmark, limiting generalizability to other European regions [47,51,52]. In Denmark, Toft et al. validated an administrative database against ground truthing to identify fast-food restaurants [51]. In this study, which also retrieved businesses using the European NACE classification scheme, authors reported sensitivity values of 82% and PPV values of 92%. In the UK, Lake et al. also validated a city council dataset against ground truthing [16]. Their validity scores were lower for sensitivity (66%) and higher for PPV (92%). This variability suggests the need to examine the accuracy of secondary data on a case-by-case basis [15]. To the best of our knowledge, our study is the first offering insight from a large Southern European setting, where there has been recent interest in food environment research [3,53].

To evaluate potential differential measurement error, previous studies also examined validity statistics by area characteristics, which could lead to confounding in the association between area-level characteristics (e.g., socioeconomic status) and food environment measures [18,48]. In this sense, we found fewer differences between area-level socioeconomic status, population density and the proportion of outlets that were correctly matched in the secondary data by using location (liberal) matching. These findings are in line with previous research, which also reported no differences by area-level deprivation [13,45,48,54,55]. Yet, we found some evidence for a systematic bias when applying a stricter matching strategy. A potential explanation for this is the small sample size, as also suggested by Paquet et al. [13]. As noted in the introduction, assessing the accuracy of secondary data across area-level socioeconomic status or urban form is warranted to avoid systematic biases in food environment studies [8,47].

In terms of the accuracy of our algorithm to correctly classify outlets by retailer type, we found very similar results regardless of the matching strategy. Although we obtained relatively high values, our results indicate some difficulties in correctly categorizing food outlets using the combination of the NACE code and the business name. This similarity in accuracy between both matching strategies, paired with the improved completeness of the dataset when using the location (liberal) matching strategy, along with the lower systematic bias with this strategy, suggests that using administrative datasets with location matching is the preferred strategy, at least in our setting.

When considering commercial vs. administrative data for assessing retail food access, previous research has shown the latter to be more accurate [15,33]. Further, administrative datasets are usually freely available data sources. This is promising for researchers, but also for public health practitioners and decision-makers. Administrative datasets can serve as valuable local surveillance data for evaluating, for instance, the impact of zoning policies (e.g., restricting fast-food outlets). On the other hand, for researchers, they can allow studying changes in the retail food environment over time. Yet, acknowledging the dataset’s accuracy and completeness is essential when using it by researchers to describe and measure the retail food environment.

Our study had some limitations that should be noted. First, our assessment of the retail food environment was limited to food outlets; therefore, food services (e.g., take-away) were not included. Second, it remains possible that observers missed food outlets. However, we trained observers to walk the entirety of street segments in every census tract, so it is unlikely that outlets were missed. Third, we did not measure any inter-rater agreement between observers.

Finally, our results may not be generalizable to other cities due to a variability in administrative datasets, but the validation procedure could be adopted by other regions to validate their own datasets.

Despite these limitations, our study presents several strengths. First, we classified food outlets based on their external appearance, but also on the in-store characteristics (based on the sales of specific products). Thereby, we unlikely misclassified any food outlet during ground-truthing. Second, the administrative dataset is from the same time point (June 2016) than when the data were collected in the field; therefore, we excluded errors due to food retail turnover over time. Third, we conducted a highly laborious record-to-record matching. Finally, this secondary dataset is freely available and updated monthly, which is a major strength, given that the retail food environment is highly dynamic.

## 5. Conclusions

We examined the completeness and accuracy of a freely-available, administrative data to examine the retail food environment in a large Southern European city like Madrid. We reported high validity measures, suggesting that administrative datasets may offer a reliable data source to measure retail food access, especially when conducting location matching. We found no systematic bias, neither in relation to area-level socioeconomic status, nor to population density with this matching strategy. While there may not be a single data source to characterize food environments, the use of previously validated secondary food outlet data is recommended.

## Figures and Tables

**Table 1:** Classification of food outlets.

Food Outlet Category	Characteristics
*Unspecialized food outlets, including outlets primarily engaged in retailing a general line of foods*	
Supermarkets	Full-line, self-service food outlets that allow the supply of a wide variety of products of daily consumption, food and non-food, without the intermediation of a person employed to serve the buyers (unless requested). This category includes both large chain, small and discount supermarkets.
Small grocers	Neighborhood stores, self-service outlets selling a variety of products and which are neither a specialized food store, a convenience store, nor a supermarket.
Convenience food stores	Outlets with a diversified product offering including food, drinks, snacks, or magazines. They usually open more than 18 hours a day, have two or fewer cash registers, and are often associated (in Spain) with gas stations.
*Specialized food outlets, including outlets primarily engaged in retailing specialized lines of food*	
Fruit & Vegetables stores	Specialized food outlet with retail sale of fresh, prepared or preserved fruits and vegetables.
Butcheries	Specialized food outlet with retail sale of fresh, frozen, or cured meat and meat products, including poultry and the retail sale of dairy products and eggs
Fishmongers	Specialized food outlet with retail sale of fresh, frozen, or cured fish and other seafood products
Bakeries	Specialized food outlet with retail sale of bread, cakes, flour confectionery and sugar confectionery
Other specialized food stores	Specialized food outlet that does not fit into any other category (e.g., gourmet food stores)

**Table 2:** Completeness of the administrative dataset by outlet matching strategy.

Measure	Liberal Matching (*n* = 101)		Strict Matching (*n* = 58)	
	Est. ^1^	95% CI ^2^	Est. ^1^	95% CI ^2^
Sensitivity	0.95	[0.89, 0.98]	0.55	[0.44, 0.64]
Positive Predictive Value	0.79	[0.70, 0.85]	0.45	[0.37, 0.54]

^1^ Est., Validity Statistic Estimate. ^2^ CI, Confidence Interval.

**Table 3:** Assessment of the systematic bias of the administrative data according to area-level socioeconomic status and population density.

Area-Level Characteristic	Liberal Matching (*n* = 101)		Strict Matching (*n* = 58)	
	Sens. ^1^	PPV ^2^	Sens. ^1^	PPV ^2^
Socioeconomic status				
Low	0.93 [0.86, 0.99]	0.92 [0.83, 1.00]	0.44 [0.26, 0.63]	0.44 [0.25, 0.63]
Middle	0.98 [0.90, 1.00]	0.63 [0.49, 0.77]	0.69 [0.40, 0.98]	0.69 [0.40, 0.98]
High	0.98 [0.91, 1.00]	0.70 [0.62, 0.78]	0.78 [0.57, 0.99]	0.78 [0.57, 0.99]
	*p* = 0.64 ^3^	*p* = 0.04 ^3^	*p* = 0.09 ^3^	*p* = 0.09 ^3^
Population density (10^3^ residents/km^2^)				
Low	0.97 [0.92, 1.00]	0.79 [0.70, 0.88]	0.71 [0.56, 0.86]	0.71 [0.56, 0.86]
Middle	0.91 [0.83, 0.99]	0.78 [0.61, 0.95]	0.35 [0.24, 0.49]	0.37 [0.24, 0.49]
High	0.95 [0.89, 1.00]	0.78 [0.64, 0.92]	0.51 [0.33, 0.69]	0.51 [0.33, 0.69]
	*p* = 0.41 ^3^	*p* = 0.99 ^3^	*p* = 0.008 ^3^	*p* = 0.008 ^3^

^1^ Sens., Sensitivity; ^2^ PPV, Positive Predictive Value. ^3^ *p*-values test the null hypothesis that sensitivity or PPV are the same in areas of low, middle and high socioeconomic status (SES) or population density.

**Table 4:** Accuracy of the algorithm to classify correctly the type of outlet in the administrative dataset, by outlet matching strategy.

Measure	Liberal Matching (*n* = 101)		Strict Matching (*n* = 58)	
	Est. ^1^	95% CI ^2^	Est. ^1^	95% CI ^2^
Percent Agreement	0.71	[0.62, 0.80]	0.77	[0.66, 0.88]
Cohen’s Kappa	0.62	[0.57, 0.66]	0.71	[0.56, 0.85]

^1^ Est., Validity Statistic Estimate; ^2^ CI, Confidence Interval.

## Supplementary Materials

The following are available online at https://www.mdpi.com/1660-4601/16/19/3538/s1, Figure S1: Example of allowable discrepancies in street names discrepancies, Table S1: Descriptive statistics of the 42 census tracts included in the street audits (Madrid, 2016), Table S2: Statistical Classification of Economic Activities in the European Community (NACE) codes and definitions and corresponding codes and definitions in the National Classification of Economic Activities (CNAE) in Spain, Table S3: List of un-matched food outlets (*n* = 24) due to discrepancies in food outlet name, Table S4: Contingency table of food outlets, by outlet type and using a liberal matching strategy (*n* = 101), Table S5: Contingency table of food outlets, by outlet type and using a strict matching strategy (*n* = 58). Table S6: Results of the log-binomial regression from where the results of Table 3 are derived.

## References

[1] Glanz K., Sallis J.F., Saelens B.E., Frank L.D. (2005). Healthy nutrition environments: Concepts and measures. Am. J. Heal. Promot..

[2] Story M., Kaphingst K.M., Robinson-O’Brien R., Glanz K. (2008). Creating healthy food and eating evironments: Policy and environmental approaches. Annu. Rev. Public Health.

[3] Bilal U., Jones-Smith J., Diez J., Lawrence R.S., Celentano D.D., Franco M. (2018). Neighborhood social and economic change and retail food environment change in Madrid (Spain): The heart healthy hoods study. Health Place.

[4] Hawkes C., Smith T.G., Jewell J., Wardle J., Hammond R.A., Friel S., Thow A.M., Kain J. (2015). Smart food policies for obesity prevention. Lancet.

[5] Caspi C.E., Sorensen G., Subramanian S.V., Kawachi I. (2012). The local food environment and diet: A systematic review. Health Place.

[6] Cobb L.K., Appel L.J., Franco M., Jones-Smith J.C., Nur A., Anderson C.A.M. (2015). The relationship of the local food environment with obesity: A systematic review of methods, study quality, and results. Obesity.

[7] Williams J., Scarborough P., Matthews A., Cowburn G., Foster C., Roberts N., Rayner M. (2014). A systematic review of the influence of the retail food environment around schools on obesity-related outcomes. Obes. Rev..

[8] Wilkins E.L., Morris M.A., Radley D., Griffiths C. (2017). Using Geographic Information Systems to measure retail food environments: Discussion of methodological considerations and a proposed reporting checklist (Geo-FERN). Heal. Place.

[9] Glanz K., Johnson L., Yaroch A.L., Phillips M., Ayala G.X., Davis E.L. (2016). Measures of retail food store environments and sales: Review and implications for healthy eating initiatives. J. Nutr. Educ. Behav..

[10] Lebel A., Daepp M.I.G., Block J.P., Walker R., Lalonde B., Kestens Y., Subramanian S.V. (2017). Quantifying the foodscape: A systematic review and meta-analysis of the validity of commercially available business data. PLoS ONE.

[11] Lytle L.A., Sokol R.L. (2017). Measures of the food environment: A systematic review of the field, 2007–2015. Heal. Place.

[12] Wilkins E., Radley D., Morris M., Hobbs M., Christensen A., Marwa W.L., Morrin A., Griffiths C. (2019). A systematic review employing the GeoFERN framework to examine methods, reporting quality and associations between the retail food environment and obesity. Health Place.

[13] Paquet C., Daniel M., Kestens Y., Léger K., Gauvin L. (2008). Field validation of listings of food stores and commercial physical activity establishments from secondary data. Int. J. Behav. Nutr. Phys. Act..

[14] Glanz K., Handy S.L., Henderson K.E., Slater S.J., Davis E.L., Powell L.M. (2016). Built environment assessment: Multidisciplinary perspectives. Popul. Health.

[15] Fleischhacker S.E., Evenson K.R., Sharkey J., Pitts S.B.J., Rodriguez D.A. (2013). Validity of secondary retail food outlet data: A systematic review. Am. J. Prev. Med..

[16] Lake A.A., Burgoine T., Greenhalgh F., Stamp E., Tyrrell R. (2010). The foodscape: Classification and field validation of secondary data sources. Health Place.

[17] Liese A.D., Colabianchi N., Lamichhane A.P., Barnes T.L., Hibbert J.D., Porter D.E., Nichols M.D., Lawson A.B. (2010). Validation of 3 food outlet databases: Completeness and geospatial accuracy in rural and urban food environments. Am. J. Epidemiol..

[18] Powell L.M., Han E., Zenk S.N., Khan T., Quinn C.M., Gibbs K.P., Pugach O., Barker D.C., Resnick E.A., Myllyluoma J. (2011). Field validation of secondary commercial data sources on the retail food outlet environment in the US. Health Place.

[19] Lo B.K., Minaker L.M., Mah C.L., Cook B. (2016). Development and testing of the Toronto nutrition environment measures survey-store. J. Nutr. Educ. Behav..

[20] Minaker L.M., Shuh A., Olstad D.L., Engler-Stringer R., Black J.L., Mah C.L. (2016). Retail food environments research in Canada: A scoping review. Can. J. Public Health.

[21] Seliske L., Pickett W., Bates R., Janssen I. (2012). Field validation of food service listings: A comparison of commercial and online Geographic Information System databases. Int. J. Environ. Res. Public Health.

[22] Murphy M., Badland H., Jordan H., Koohsari M., Giles-Corti B. (2018). Local food environments, suburban development, and BMI: A mixed methods study. Int. J. Environ. Res. Public Health.

[23] Feng X., Astell-Burt T., Badland H., Mavoa S., Giles-Corti B. (2018). Modest ratios of fast food outlets to supermarkets and green grocers are associated with higher body mass index: Longitudinal analysis of a sample of 15,229 Australians aged 45 years and older in the Australian National Liveability Study. Health Place.

[24] Diez J., Bilal U., Cebrecos A., Buczynski A., Lawrence R.S., Glass T., Escobar F., Gittelsohn J., Franco M. (2016). Understanding differences in the local food environment across countries: A case study in Madrid (Spain) and Baltimore (USA). Prev. Med..

[25] Flavián C., Haberberg A., Polo Y. (2002). Food retailing strategies in the European union. A comparative analysis in the UK and Spain. J. Retail. Consum. Serv..

[26] Díez J., Bilal U., Franco M. (2018). Unique features of the Mediterranean food environment: Implications for the prevention of chronic diseases Rh: Mediterranean food environments. Eur. J. Clin. Nutr..

[27] Hees S., Horstman K., Jansen M., Ruwaard D. (2017). Photovoicing the neighbourhood: Understanding the situated meaning of intangible places for ageing-in-place. Health Place.

[28] Pinho M., Mackenbach J., Oppert J.-M., Charreire H., Bárdos H., Rutter H., Compernolle S., Beulens J., Brug J., Lakerveld J. (2019). Exploring absolute and relative measures of exposure to food environments in relation to dietary patterns among European adults. Public Health Nutr..

[29] Svastisalee C.M., Nordahl H., Glümer C., Holstein B.E., Powell L.M., Due P. (2011). Supermarket and fast-food outlet exposure in Copenhagen: Associations with socio-economic and demographic characteristics. Public Health Nutr..

[30] Achón M., Serrano M., García-González Á., Alonso-Aperte E., Varela-Moreiras G. (2017). Present food shopping habits in the Spanish adult population: A cross-sectional study. Nutrients.

[31] Odoms-Young A.M., Zenk S., Mason M. (2009). Measuring food availability and access in African-American communities. Am. J. Prev. Med..

[32] Rummo P.E., Gordon-Larsen P., Albrecht S.S. (2015). Field validation of food outlet databases: The Latino food environment in North Carolina, USA. Public Health Nutr..

[33] Gomez-Lopez I.N., Clarke P., Hill A.B., Romero D.M., Goodspeed R., Berrocal V.J., Vinod Vydiswaran V.G., Veinot T.C. (2017). Using social media to identify sources of healthy food in urban neighborhoods. J. Urban Heal..

[34] Sharkey J.R., Horel S. (2008). Neighborhood socioeconomic deprivation and minority composition are associated with better potential spatial access to the ground-truthed food environment in a large rural area. J. Nutr..

[35] Mendez D.D., Kim K.H., Hardaway C.R., Fabio A. (2016). Neighborhood racial and socioeconomic disparities in the food and alcohol environment: Are there differences by commercial data sources?. J. Racial Ethn. Heal. Disparities.

[36] Carré H. (2008). Statistical Classification of Economic Activities in the European Community.

[37] Bilal U., Díez J., Alfayate S., Gullón P., Del Cura I., Escobar F., Sandín M., Franco M. (2016). Population cardiovascular health and urban environments: The heart healthy hoods exploratory study in Madrid, Spain. BMC Med. Res. Methodol..

[38] Cebrecos A., Domínguez-Berjón M.F., Duque I., Franco M., Escobar F. (2018). Geographic and statistic stability of deprivation aggregated measures at different spatial units in health research. Appl. Geogr..

[39] Sureda X., Bilal U., Fernández E., Valiente R., Escobar F.J., Navas-Acien A., Franco M. (2018). Second-hand smoke exposure in outdoor hospitality venues: Smoking visibility and assessment of airborne markers. Environ. Res..

[40] Glanz K., Sallis J.F., Saelens B.E., Frank L.D. (2007). Nutrition Environment Measures Survey in Stores (NEMS-S). Development and Evaluation. Am. J. Prev. Med..

[41] Honeycutt S., Davis E., Clawson M., Glanz K. (2010). Training for and dissemination of the Nutrition Environment Measures Surveys (NEMS). Prev. Chronic Dis..

[42] Open Data Kit. https://opendatakit.org/.

[43] Oliver M.N., Matthews K.A., Siadaty M., Hauck F.R., Pickle L.W. (2005). Geographic bias related to geocoding in epidemiologic studies. Int. J. Health Geogr..

[44] Gullón P., Bilal U., Cebrecos A., Badland H.M., Galán I., Franco M. (2017). Intersection of neighborhood dynamics and socioeconomic status in small-area walkability: The heart healthy hoods project. Int. J. Health Geogr..

[45] Burgoine T., Harrison F. (2013). Comparing the accuracy of two secondary food environment data sources in the UK across socio-economic and urban/rural divides. Int. J. Health Geogr..

[46] Caspi C.E., Friebur R. (2016). Modified ground-truthing: An accurate and cost-effective food environment validation method for town and rural areas. Int. J. Behav. Nutr. Phys. Act..

[47] Wilkins E.L., Radley D., Morris M.A., Griffiths C. (2017). Examining the validity and utility of two secondary sources of food environment data against street audits in England. Nutr. J..

[48] Daepp M.I.G., Black J. (2017). Assessing the validity of commercial and municipal food environment data sets in Vancouver, Canada. Public Health Nutr..

[49] Black C., Moon G., Baird J. (2014). Dietary inequalities: What is the evidence for the effect of the neighbourhood food environment?. Heal. Place.

[50] Charreire H., Casey R., Salze P., Simon C., Chaix B., Banos A., Badariotti D., Weber C., Oppert J.M. (2010). Measuring the food environment using geographical information systems: A methodological review. Public Health Nutr..

[51] Toft U., Erbs-Maibing P., Glümer C. (2011). Identifying fast-food restaurants using a central register as a measure of the food environment. Scand. J. Public Health.

[52] Svastisalee C.M., Holstein B.E., Due P. (2012). Validation of presence of supermarkets and fast-food outlets in Copenhagen: Case study comparison of multiple sources of secondary data. Public Health Nutr..

[53] Díez J., Cebrecos A., Rapela A., Borrell L.N., Bilal U., Franco M. (2019). Socioeconomic inequalities in the retail food environment around schools in a southern European context. Nutrients.

[54] Clary C.M., Kestens Y. (2013). Field validation of secondary data sources: A novel measure of representativity applied to a Canadian food outlet database. Int. J. Behav. Nutr. Phys. Act..

[55] Rossen L.M., Pollack K.M., Curriero F.C. (2012). Verification of retail food outlet location data from a local health department using ground truthing and remote-sensing technology: Assessing differences by neighborhood characteristics. Health Place.

