# Supplementary material for: Assessing the Retail Food Environment in Madrid: An Evaluation of Administrative Data against Ground Truthing
Source: Int J Environ Res Public Health. 2019 Sep 21;16(19):3538. doi: 10.3390/ijerph16193538 (PMC6801710; doi:10.3390/ijerph16193538)
Supplement: Supplementary file 1 [file ijerph-16-03538-s001.pdf]

SUPPLEMENTARY MATERIAL

Figure S1. Example of allowable street names discrepancies. This figure shows an example of a food outlet listed in the administrative dataset as being located in 'San Narciso Street' (on the right). that was recorded during ground-truthing to be located on 'Esfinge Street' (on the left). We conducted this manual process of examining discrepancies using Google Street View. Image source: Google Street View

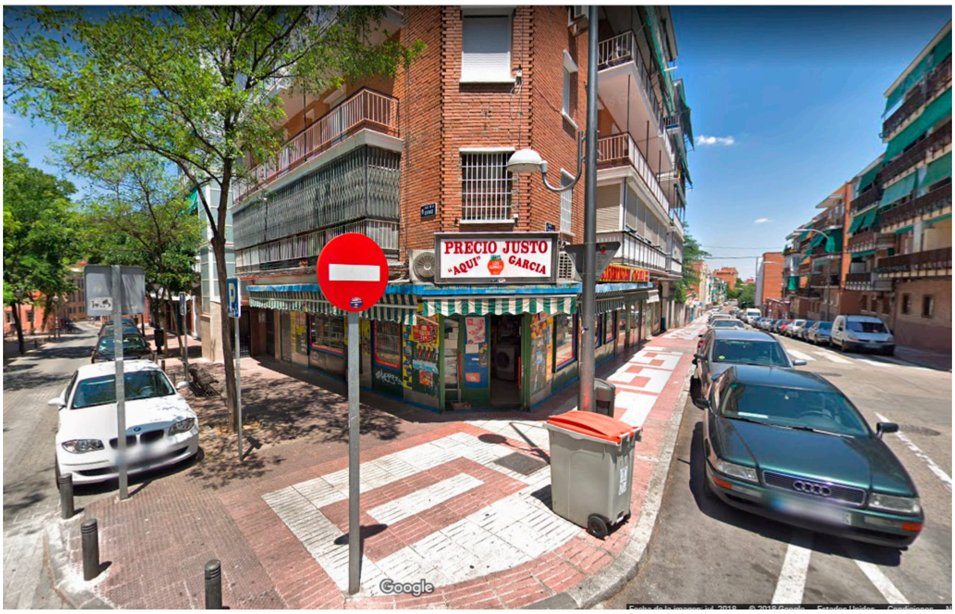

Table S1. Descriptive statistics of the 42 census tracts included in the street audits (Madrid, 2016)

Census Tract	Area-level SES[1]	Low Education (%)	% of population (age ≥65)	% of foreign-born	Population density (pop/km^2)	Retail density (retailers/km^2)	Land area (km^2)
1	0.49	16.89	18.44	30.77	32731.94	1238.94	0.03
2	0.47	13.40	16.27	29.93	38541.94	1812.05	0.03
3	0.68	15.74	18.76	13.82	44104.48	316.73	0.03
4	0.42	15.08	20.89	20.57	39496.77	485.41	0.04
5	0.73	12.36	16.35	12.75	42174.88	114.45	0.03
6	13.92	5.77	25.84	10.63	48290.04	176.99	0.03
7	12.82	8.58	24.46	17.85	40539.74	810.79	0.02
8	18.40	6.81	22.25	22.11	20595.24	303.51	0.07
9	14.56	6.69	26.80	13.40	13222.39	172.19	0.14
10	17.48	4.41	21.40	14.72	9278.95	6.33	0.16
11	-0.18	18.53	17.98	37.89	45726.18	360.31	0.03
12	-0.22	24.32	15.41	26.73	32776.81	171.79	0.06
13	10.21	10.77	19.16	22.07	37623.68	568.07	0.02
14	10.79	10.58	24.23	21.63	36603.89	387.16	0.03
15	0.26	18.35	25.53	12.76	30204.03	260.88	0.03
16	16.56	2.33	2.70	7.62	7395.24	26.84	0.34
17	11.65	7.40	25.18	15.33	35385.75	619.90	0.04
18	14.36	5.12	13.94	13.35	4303.60	0.00	0.35
19	-0.74	32.57	20.77	23.78	22565.16	89.12	0.06
20	-0.65	26.56	29.77	21.28	37135.86	218.63	0.03
21	-11.15	28.53	21.94	29.26	40390.71	254.67	0.02
22	-0.91	27.12	22.24	27.94	26344.27	168.10	0.08
23	-0.79	21.83	21.29	25.80	35399.16	379.78	0.05
24	-12.21	32.43	16.35	48.82	42808.95	535.90	0.03
25	-13.33	30.82	15.10	34.76	42179.87	384.55	0.04
26	-13.16	30.74	19.28	30.58	44701.12	246.29	0.02
27	-0.37	31.93	26.05	10.34	26772.57	41.64	0.05
28	-0.26	28.05	33.12	15.70	35352.14	166.49	0.02
29	0.30	15.24	23.31	20.72	27504.61	349.51	0.07
30	0.40	18.36	26.92	14.62	29021.18	336.25	0.04
31	-0.09	24.03	26.09	14.60	21512.82	137.40	0.05
32	-0.19	23.41	17.55	13.34	16447.13	22.32	0.13
33	-22.33	40.66	16.47	46.34	38594.41	101.51	0.05
34	-10.68	31.93	15.33	34.97	34244.11	206.91	0.04
35	-0.81	31.68	19.64	19.05	44769.25	227.45	0.03
36	-0.31	15.09	19.46	20.73	27657.39	93.60	0.04
37	-0.56	38.56	24.88	28.27	24789.71	666.08	0.03
38	-13.33	38.56	27.48	25.33	19634.09	180.50	0.05
39	-0.98	42.71	25.10	15.59	40346.41	0.00	0.02
40	-0.53	26.91	23.55	20.80	34207.49	369.27	0.06
41	-0.40	24.04	16.44	24.23	16943.91	250.18	0.09
42	15.53	10.52	10.55	12.60	5330.93	0.00	0.28
**Mean (SD)**	1.33 (9.42)	20.84 (11.05)	20.82 (5.54)	21.99 (9.42)	30801.16 (11825.84)	315.68 (339.99)	0.07 (0.08)
**Median (IQR)**	-0.19 (-0.78, 10.20)	20.18 (10.77, 30.74)	21.09 (16.46, 25.10)	20.77 (14.59, 27.93)	34225.80 (22565.16, 40346.41)	236.87 (114.45, 379.77)	0.04 (0.03, 0.05)
**Min**	-22.33	2.33	2.70	7.62	4303.60	0.00	0.02
**Max**	18.40	42.71	33.12	48.82	48290.04	1812.05	0.35

[1] *Area-level socioeconomic status was measured using a composite index, developed by Gullon et al [45], which is made up of seven indicators (low education, high education, part-time work, temporary work, manual work, unemployment, and average housing prices).*

Table S2. Statistical Classification of Economic Activities in the European Community (NACE) codes and definitions and corresponding codes and definitions in the National Classification of Economic Activities (CNAE) in Spain

NACE[1] code	NACE definition	CNAE[2] code and definition
47.1	Retail sale in non-specialized stores	
47.11	Retail sale in non-specialized stores[3] with food. beverages or tobacco predominating.	47.11.01 – 'autoservicio'<br>47.11.02 – 'grandes superficies'<br>47.11.03 – 'tienda de conveniencia. 24h'
47.2	Retail sale in specialized stores	
47.21	Retail sale of fruits and vegetables in specialized stores. including the retail sale of fresh. prepared and preserved fruits and vegetables	47.21.01; 47.21.02 – 'comercio de frutas y hortalizas'
47.22	Retail sale of meat and meat products (including poultry)	47.22.01 – 'carnicería'<br>47.22.02 – 'charcutería'<br>47.22.03 – 'carnicería-charcutería'<br>47.22.04 – 'carnicería-salchichería'<br>47.22.05 and 47.22.06 – 'aves, huevos y caza'<br>47.22.07 – 'casquería'
47.23	Retail sale of fish. crustaceans and molluscs	47.23.01 and 47.23.02 '–pescado y mariscos'<br>47.23.03 – 'bacalao'
47.24	Retail sale of bread. cakes. flour confectionery and sugar confectionery	47.24.01 and 47.24.02 – 'pan. productos de panadería y bollería'<br>47.24.03 - 47.24.05 'pastelería. confitería. Repostería'
47.29	Other retail sale of food in specialized stores not else classified	47.29.01 – 'herbolario'<br>47.29.02 – 47.29.04 'heladería'<br>47.29.05 – 'congelados'<br>47.29.06 – 'golosinas'<br>47.29.07 – 'frutos secos'

[1] *NACE is the acronym for "Nomenclature statistique des activités économiques dans la Communauté européenne"*
[2] *CNAE is the acronym for "Clasificación Nacional de Actividades Económicas"*
[3] *NACE classification does not differentiate between unspecialized retailers and includes the entire range of large chain, small independent and discount supermarkets together with convenience stores*

Table S3. List of un-matched food outlets (N=24) due to discrepancies in food outlet names

Food outlet name recorded during ground-truthing	Food outlet name registered on the administrative dataset
Panaderia	Prensa y alimentaci0n vicente
Alimentacion R. Garcia	Garcia rubio jose ramon
Bazar	Alimentacion xiaoli shan
Spass dia 365	Alimentacion
Tahona castellana	Croisanteria alonso (antes felipe godoy)
Arganium	Rotulo no informado
Frutas y verduras emi	Violeta
Bocata del barrio	Sin indicar
Frutas y verduras	Alimentacion
Comercial madrid	Alimentacion
Claudia	Fruteria mohamed
Superconver	Hiper dis'frutas
Super euro	Alimentacion suyan
Fruteria la plaza	Rotulo no informado
Alimentacion y bazar	Sin determinar
Alimentacion zhou	(en chino)
Herbolario soleil	Centro de estetica
Alimentos asiaticos	Sin rotulo
Sin nombre	Frutas y verduras youssef
Carniceria fruteria amina	Alimentacion khalid morabet
Frutas verduras yanira	R0tulo no informado
Fruteria	Snapo
Natur fruta	Tienda de alimentacion
Alimentacion grupo t	Sr

Table S4. Contingency table of food outlets, by outlet type as measured by ground-truthing and using the administrative dataset and using a liberal matching strategy (N=101)

	Administrative dataset							
Ground-truthing	supermarkets	small grocers	convenience	F&V stores	butcheries	fishmongers	bakeries	other
supermarkets	**13**	5	0	1	0	0	0	0
small grocers	0	**31**	0	2	0	0	0	4
convenience	0	1	**1**	0	0	0	0	1
F&V stores	0	4	0	**7**	0	0	0	4
butcheries	0	2	0	0	**5**	0	0	0
fishmongers	0	0	0	0	0	**2**	0	0
bakeries	0	3	0	1	0	0	**7**	0
other	0	1	0	0	0	0	0	**6**

Table S5. Contingency table of food outlets, by outlet type as measured by ground-truthing and using the administrative dataset and using a strict matching strategy (N=58)

	Administrative dataset							
Ground-truthing	supermarkets	small grocers	convenience	F&V stores	butcheries	fishmongers	bakeries	other
supermarkets	**11**	3	0	0	0	0	0	0
small grocers	0	**16**	0	1	0	0	0	2
convenience	0	0	**1**	0	0	0	0	0
F&V stores	0	2	0	**4**	0	0	0	0
butcheries	0	2	0	0	**2**	0	0	0
fishmongers	0	0	0	0	0	**2**	0	0
bakeries	0	1	0	1	0	0	**6**	0
other	0	1	0	0	0	0	0	**3**

Table S6. Results of the log-binomial regression from where the results of Table 3 are derived.

	**Liberal matching (N=101)**		**Strict matching** (N=58)	
	**Sens PR (95% CI)**	**PPV PR (95% CI)**	**Sens PR (95% CI)**	**PPV PR (95% CI)**
**Socioeconomic status**				
Low	1 (Ref.)	1 (Ref.)	1 (Ref.)	1 (Ref.)
Middle	1.04 [0.94, 1.14]	0.72 [0.57, 0.91]	1.30 [0.74, 2.27]	1.30 [0.74, 2.27]
High	1.04 [0.95, 1.14]	0.84 [0.73, 0.95]	1.61 [1.02, 2.54]	1.61 [1.02, 2.54]
**Population density (10^3 residents/km^2)**				
Low	1 (Ref.)	1 (Ref.)	1 (Ref.)	1 (Ref.)
Middle	0.94 [0.85, 1.03]	0.99 [0.78, 1.25]	0.55 [0.37, 0.80]	0.55 [0.37, 0.80]
High	0.99 [0.94, 1.07]	0.99 [0.81, 1.20]	0.84 [0.57, 1.25]	0.84 [0.57, 1.25]
